# A comparative study of transgender women accessing HIV testing via face-to-face and telemedicine services in Chiang Mai, Thailand during the COVID-19 pandemic and their risk of being HIV-positive

**DOI:** 10.1186/s12889-023-17124-2

**Published:** 2023-11-04

**Authors:** Nontiya Homkham, Natthaporn Manojai, Pongpeera Patpeerapong, Rattawit Apiputthipan, Pimwarat Srikummoon, Unyamanee Kummaraka, Phisanu Chiawkhun, Apinut Rankantha, Patrinee Traisathit

**Affiliations:** 1 Faculty of Public Health, Thammasat University, Pathumthani, Thailand; 2 Mplus Foundation, Chiang Mai, Thailand; 3 Department of Statistics, Faculty of Science, Chiang Mai University, Chiang Mai, Thailand

**Keywords:** HIV testing, Telemedicine service, Face-to-face service, COVID-19, Transgender women

## Abstract

**Background:**

Due to the restricted availability of health services in Thailand, there are still some transgender women (TGW) who do not have access to HIV counseling and testing. Telehealth, which is accessible to individuals who are reluctant to undergo face-to-face interviewing, played an especially important role during the COVID-19 epidemic. The objectives of this study are to compare the characteristics, pattern of accessing HIV testing, and the HIV-positive rates of TGW between the face-to-face and telemedicine services.

**Methods:**

We conducted a cross-sectional study to compare the access to HIV testing and the HIV-positive rates among TGW via face-to-face service and telemedicine services and examined the influence of potential associated factors on the risk of being HIV-positive.

**Results:**

Of the 637 TGW participants, 26 (4.1%) were HIV-positive. Accessing the telemedicine service increased in the third and fourth COVID-19 waves (28.1% in the first and second vs. 71.9% in the third and fourth). There was no difference in the risk of being HIV-positive between the types of service. Having sex work experience (adjusted odds ratio (aOR) = 5.92; 95% confidence interval (CI): 1.57–22.30) and either never having been or tested more than 1 year ago were independently significantly associated with a higher risk of being HIV-positive (aOR = 4.05; 95% CI: 1.11–14.77).

**Conclusion:**

The telemedicine service became more popular among TGW during the COVID-19 pandemic and was not related to a higher risk of being HIV-positive. Moreover, it proved to be an effective alternative channel to access HIV testing, especially for intravenous drug users. Sex work experience and irregular HIV testing are key risk factors for HIV infection in TGW seeking either the telemedicine or face-to-face service.

## Introduction

To end the AIDS epidemic by the end of 2030, the Joint United Nations Programme on HIV and AIDS (UNAIDS) launched the 95-95-95 targets in 2014 [1]. Thailand, with an estimated HIV prevalence of 1.0% in adults [2], pledged to achieve these targets and is well on the way to accomplishing this; in 2021, 94% of people with HIV were aware of their status, 86% of people living with HIV (PLHIV) who know their status were receiving antiretroviral therapy (ART), and 84% of PLHIV had a suppressed viral load. However, HIV infection, prevention, and treatment among transgender (TG) people remain important issues. The World Health Organization (WHO) stated that TG individuals are around 13 times more likely to become HIV-positive than other adults [3] and the HIV prevalence among TG women (TGW) aged 15–49 years old is 19 times greater than other women in the same age group [4]. According to the results of a recent meta-analysis, the overall standardized HIV prevalence rate for TGW across various countries and age groups is 19.9%, which highlights the persistence of this issue even during the COVID-19 epidemic [5]. The HIV prevalence among TGW in Thailand decreased from 12.7% to 2014 [6] to 4.2% in 2020 [7]. Although the prevalence of HIV has generally decreased in Thailand, HIV testing of TG individuals is still of concern.

The COVID-19 pandemic negatively affected Thailand; many healthcare providers suspended or restricted their services and transportation became difficult due to the fear of contracting the disease. These factors along with others (i.e., access to HIV prevention and treatment services, HIV testing, or inadvertently HIV disclosure and stigmatization from being infected with COVID-19) have slowed the process of HIV eradication [8–10]. The Institute for HIV Research and Innovation commented that the COVID-19 pandemic reduced the amount of HIV testing in Thailand in 2020 and whether testing for COVID-19, HIV, or AIDS, the key is involving the community and civil society organizations. With empowerment and support, the latter has played an important role in helping to design a system for accessible medical services [11]. Therefore, online [12] and mail delivery [13] have become optional channels for reaching out to target populations for HIV testing.

The MPlus Medical Technology Clinic in Chiang Mai, Thailand works on HIV prevention and human rights issues affecting homosexual people, TGW, and sex workers. This organization provides HIV voluntary counseling and testing (VCT) through community-based organizations, including free HIV testing, sexually transmitted infection (STI) screening, sexual health counseling, and community outreach. In addition to services in stand-alone centers, MPlus provides mobile VCT (MVCT) to rural areas of Chiang Mai and has been reaching even more of the target group through several activities while offering free HIV testing, hormone-level checks, knowledge about hormone therapy for TGW, and HIV prevention advice [14]. Since telemedicine has been playing an important role since the beginning of the COVID-19 pandemic, MPlus offers telemedicine services and online consulting via social media channels in addition to the face-to-face service at the stand-alone center or an MVCT. TGW living in Chiang Mai or nearby provinces who want to take an HIV test or partake in another service at the clinic can make an online reservation before visiting it or a nearby MPlus-affiliated hospital.

Based on the outcomes of previous studies, we hypothesized that the telemedicine service could be beneficial for recruiting more of the target population (TGW) for HIV testing during the COVID-19 pandemic. Therefore, the objective of this study was to explore the pattern of accessing HIV testing and any differences between the characteristics of TGW clients based on the type of service (i.e., face-to-face or telemedicine), to identify whether the type of service was influential, and to determine the risk of being HIV positive while adjusting for potentially associated factors according to the type of service.

## Methods

### Participants and setting

TGW aged ≥ 18 years old with an unknown HIV status who accessed one of the services provided by the MPlus Foundation: face-to-face (both stand-alone center and MVCT service) or telemedicine during the COVID-19 pandemic from October 2020 to January 2022 were included in the study. All of the participants underwent sexual health counseling and voluntary HIV testing.

### Data collection and measurements

The socio-demographic information of the study participants comprised age (defined in ranges), relationship status (single/with a partner), history of taking pre-exposure prophylaxis (PrEP) before HIV testing (yes/no), history of taking post-exposure prophylaxis (PEP) before HIV testing (yes/no), history of drug abuse (yes/no), history of injected drug use (yes/no), sex worker experience (yes/no), history of HIV testing, the period between exposure and testing for HIV, and other STIs (e.g., syphilis (non-reactive/reactive)), HIV test results (negative/positive), reasons for accessing the service at MPlus (want to check their hormone levels, a follow-up PrEP visit, received information about the services at MPlus, exposure to a high risk of HIV infection, recommended by friends, or want to know their HIV status), and channels of information and promotion of HIV testing and services at MPlus.

The history of HIV testing was obtained by interviewing the participants categorized into 5 groups: (1) no prior HIV testing, (2) more than 1 year since HIV testing, (3) HIV testing within the previous 6–12 months, (4) HIV testing within the previous 3–6 months, and (5) HIV testing within the previous 3 months. The window period for HIV testing was defined as 14 days after HIV exposure [15].

Since this study was conducted during the COVID-19 pandemic, we thus categorized the time period of accessing the services into 4 periods: (1) the first wave (12 January 2020 to 30 November 2020), (2) the second wave (1 December 2020 to 31 March 2021), (3) the third wave (1 April 2021 to 31 December 2021), and (4) the fourth wave (since 1 January 2022) [16].

### HIV and STI testing services

**Fig. 1:**
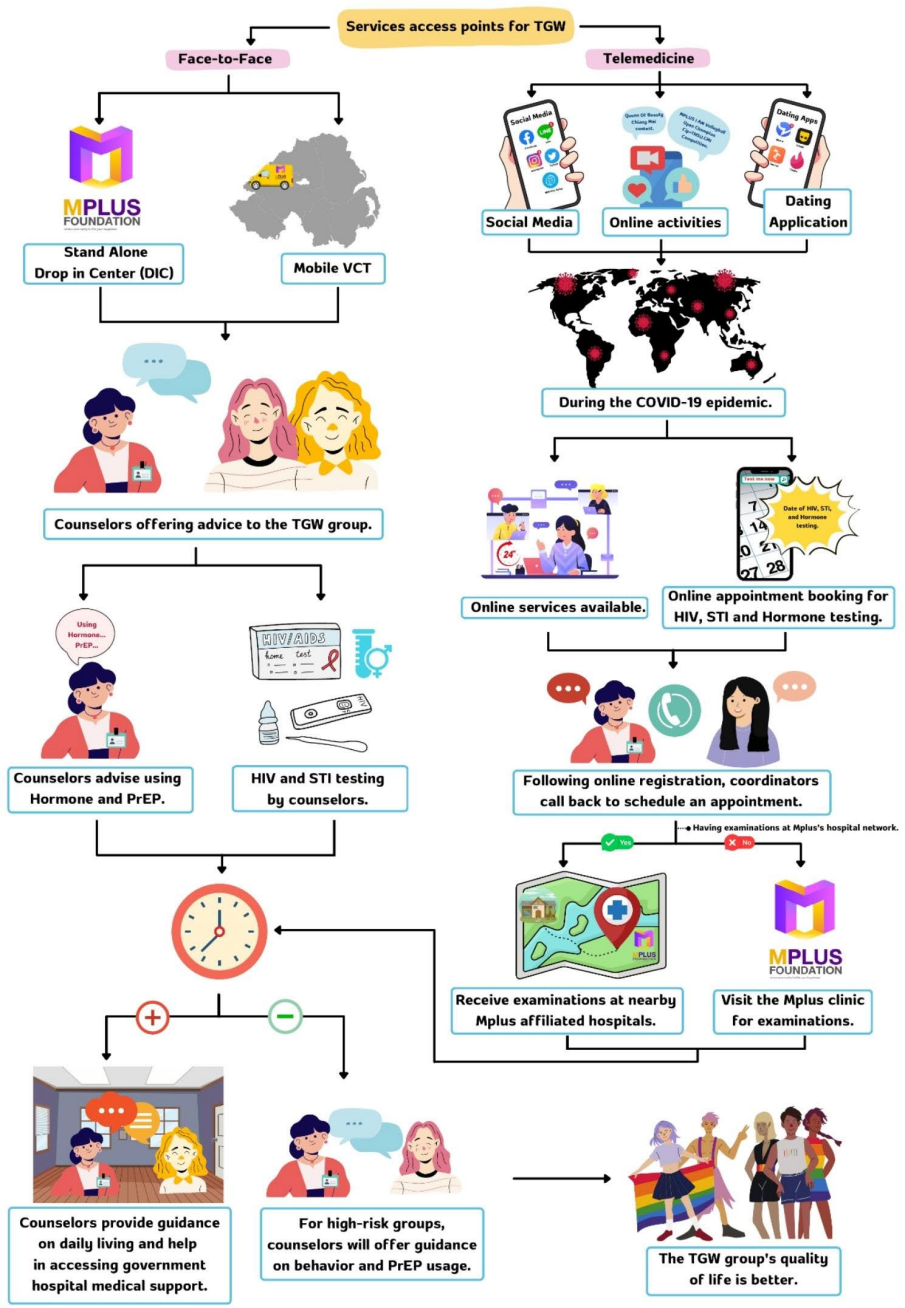
Schematics of the face-to-face and telemedicine services provided by the MPlus Foundation

HIV testing was performed using rapid test kits (Determine HIV-1/2), which provide results within 20 min. The participants with an HIV-positive test result were double-checked with other rapid test kits (Colloidal Gold Device and SD Bioline HIV 1/2 3.0). They were also screened for incident syphilis infection using SD BIOLINE Syphilis 3.0 rapid test kits. Both the face-to-face and telemedicine services are free of charge. The procedures for the face-to-face and the telemedicine service offered by MPlus Foundation are presented in Fig. 1.

The face-to-face service is provided at the MPlus stand-alone drop-in center and MVCT service. The face-to-face counseling and testing services were conducted on-site by well-trained MPlus staff. The test results were reported directly to the participants. Those who had an HIV-negative test result were advised about their individual preferences and levels of risk. Those with high-risk behavior were also advised to take PrEP in combination with using condoms and received telephone reminders to undergo follow-up testing. For the participants who had an HIV-positive test result, the counselors advised them on how to live with HIV and transferred them to patient care at a government hospital. The MPlus staff who provided the counseling were well-trained and had received certificates from the Department for Disease Control, Thailand and the USAIDS partnership. To comply with the guidelines for preventing the spread of COVID-19 in Thailand, the counselor and participant were in a private room, wearing masks, and staying more than 1 m apart for the entire process.

The telemedicine service was launched during the Covid-19 pandemic in October 2020. This service provides online counseling about HIV and STI prevention via online platforms such as Facebook, Line, and Twitter, among others. Moreover, reservations for on-site HIV, STI, and hormone testing can also be made. After making an appointment, a member of the MPlus staff contacts the client and provides information about the available date and time for on-site HIV, STI, and/or hormone testing at a nearby MPlus-affiliated hospital or the MPlus clinic if an appointment at the former is not available. The test results are confidentially sent back to the MPlus staff who then reported them to the participant via a phone call. Counseling and advice after HIV testing are also provided via a phone call or face-to-face.

### Sample size calculation

The sample size was calculated based on the following formula [17]:

$$n = \frac{{Z_{\alpha /2}^2 \cdot P(1 - P)}}{{{e^2}}}$$

where *n* is the required sample size, *e* (the accepted error in this study) was set as 0.02, ${Z}_{\alpha /2}$ was set as 1.96 for the 95% confidence interval (CI) (i.e., $\alpha$ = 0.05), and $P$ (the prevalence of HIV-positive cases among TGW) was set as 5.5% based on the estimated HIV prevalence among TGW and men who have sex with men (MSM) in Thailand reported in a previous study [14]. Thus, at least 500 participants were required for the study.

### Statistical analysis

The participants’ demographic characteristics as well as their syphilis infection and HIV results were summarized using descriptive statistics. Characteristics and information about accessing HIV testing by the TGW individuals via the face-to-face or telemedicine service were compared using Fisher’s exact tests for categorical variables and Wilcoxon rank-sum tests for continuous variables. The risk of being HIV-positive among TGW was examined by using binary logistic regression. The potentially associated factors with *P*-values of less than 0.25 in the univariable analyses [18] (i.e., sex worker experience, prior experience of HIV testing, the STI result, and a history of drug abuse) were included in a multivariable analysis with backward elimination. In addition, since we hypothesized that the telemedicine service could be beneficial for recruiting more TGW for HIV testing during the COVID-19 pandemic [13], the type of HIV testing service (face-to-face or telemedicine) was also retained as an adjustment factor in the multivariable models even though it attained a *P*-value higher than 0.25. Subsequently, the full model for the multivariable analysis included sex worker experience, prior experience with HIV testing, the result of STI testing, a history of drug abuse, and the HIV testing service (face-to-face or telemedicine). Finally, the best-fitting logistic regression model was determined by using Pearson’s Chi-square and Hosmer-Lemeshow goodness-of-fit tests, and the Bayesian Information Criterion (BIC). All of the analyses were performed using Stata version 15.

## Results

### Socio-demographic characteristics

The data used in the analyses were obtained from 637 TGW individuals who accessed either the face-to-face service (260 individuals (40.8%)) or the telemedicine service (377 individuals (59.2%)) at the MPlus Foundation in Chiang Mai, Thailand during the COVID-19 pandemic from October 2020 to January 2022. The socio-demographic characteristics of HIV testing, including age, relationship status, history of taking PrEP, history of taking PEP, history of drug abuse, sex worker experience, the window period for HIV testing, and the source of hearing about the promotions and services at MPlus were compared between the telemedicine and face-to-face groups (Table 1).

**Table 1:** Characteristics of the transgender women study population

Characteristic	Total (N = 637)	Telemedicine (n = 260)	Face-to-Face (n = 377)	*P*-value
	n (%)	n (%)	n (%)	
Age (years old)				0.415^a^
< 20	138 (21.7)	55 (21.2)	83 (22.0)	
20–24	238 (37.4)	97 (37.3)	141 (37.4)	
25–29	129 (20.2)	60 (23.1)	69 (18.3)	
> 30	132 (20.7)	48 (18.4)	84 (22.3)	
Median [Interquartile range]	23 [20–28]	23 [20–28]	23 [20–29]	
Relationship status				0.047^a*^
Single	303 (73.9)	100 (68.0)	203 (77.2)	
With a partner	107 (26.1)	47 (32.0)	60 (22.8)	
NA	227	113	114	
History of taking PrEP before HIV testing				0.256^a^
No	282 (70.5)	99 (66.9)	183 (72.6)	
Yes	118 (29.5)	49 (33.1)	69 (27.4)	
NA	237	112	125	
History of taking PEP before HIV testing				0.403^a^
No	329 (83.3)	119 (80.9)	210 (84.7)	
Yes	66 (16.7)	28 (19.1)	38 (15.3)	
NA	242	113	129	
History of drug abuse				0.147^a^
No	360 (90.9)	138 (93.9)	222 (89.2)	
Yes	36 (9.1)	9 (6.1)	27 (10.8)	
NA	241	113	128	
Sex worker experience				0.776^a^
No	326 (83.8)	121 (82.9)	205 (84.4)	
Yes	63 (16.2)	25 (17.1)	38 (15.6)	
NA	248	114	134	
HIV-tested during the window periods				0.831^b^
No	614 (96.4)	250 (96.1)	364 (96.6)	
Yes	23 (3.6)	10 (3.9)	13 (3.5)	
Channels for obtaining information about HIV testing or services from MPlus				0.644^b^
Recommended by friends	200 (44.9)	77 (44.5)	123 (45.1)	
Staff at MPlus	142 (31.8)	59 (34.1)	83 (30.4)	
Online channels	70 (15.7)	27 (15.6)	43 (15.7)	
Self-seeking/other sources	34 (7.6)	10 (5.8)	24 (8.8)	
NA	191	87	104	

Abbreviations: NA, not applicable; PrEP, pre-exposure prophylaxis; PEP, post-exposure prophylaxis

^a^ Fisher’s exact test; ^b^ Wilcoxon rank-sum test

^*^P-value < 0.05

The median age was 23 (interquartile range (IQR): 20–28) years of age, which was almost the same for both groups (23 (20–28) for the telemedicine group and 23 (20–29) for the face-to-face group). The channels through which the participants obtained information about HIV testing or services were similar for both groups (*P* = 0.644). The most common channels for obtaining information about HIV testing or other MPlus services was recommendation by friends (44.9%) followed by staff at MPlus (31.8%). There was no significant difference in the history of taking PrEP before HIV testing, the history of taking PEP before HIV testing, the history of drug abuse, sex worker experience, and the window period for HIV testing between the groups. However, those in a relationship were more likely to access the telemedicine service than the face-to-face service (32.0% versus 22.8%; *P* = 0.047).

### Comparison between the types of service for HIV testing

**Fig. 2:**
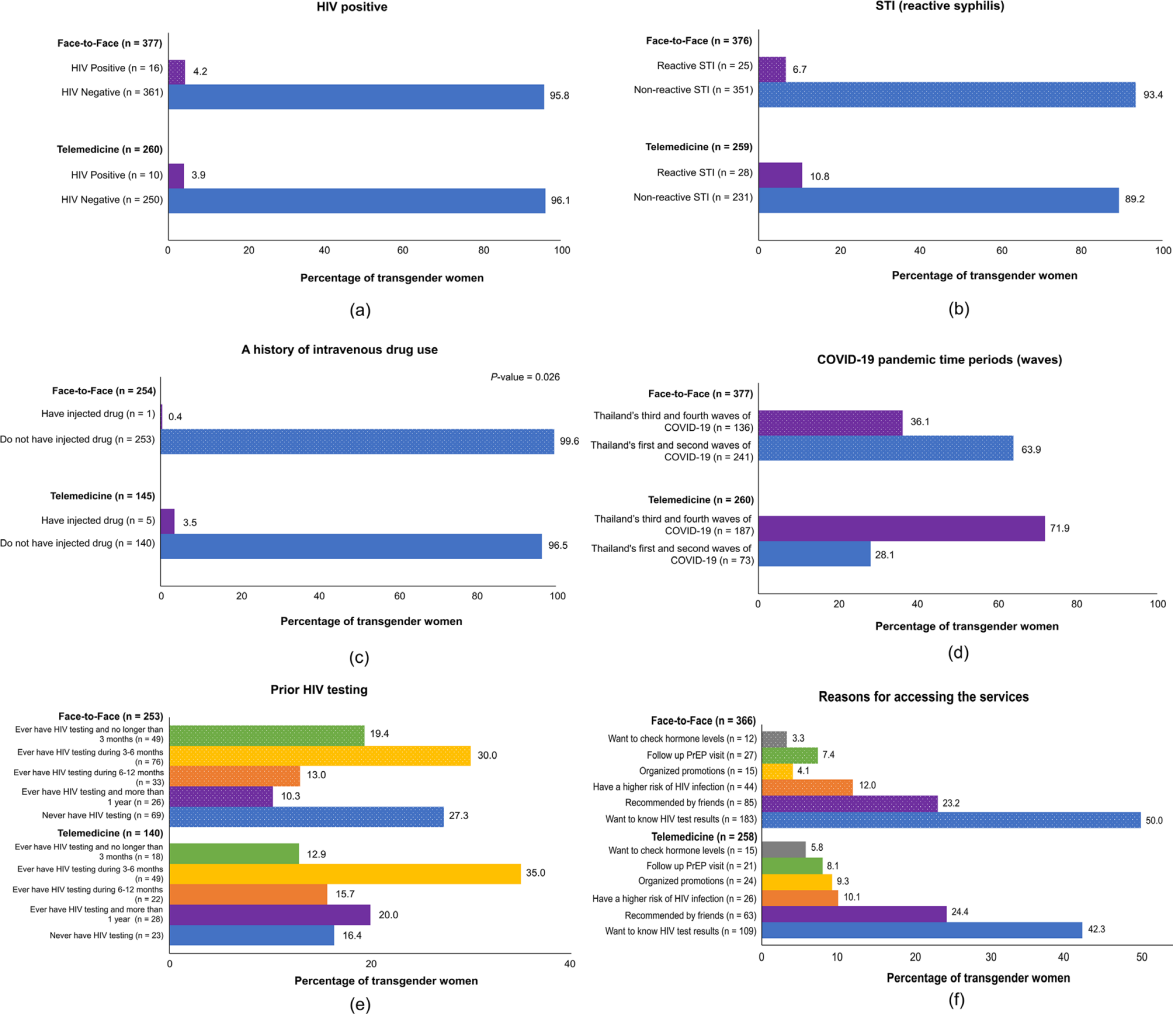
Comparison between the types of service for HIV testing among TGW who accessed the HIV testing service from the MPlus Foundation

Of the 637 TGW individuals, 26 (4.1%) were HIV-positive, including 10 (3.9%) in the telemedicine group and 16 (4.2%) in the face-to-face group (*P* = 0.842) (Fig. 2a). The proportions who had reactive STI were similar (10.8% for the telemedicine group versus 6.7% for the face-to-face group; *P* = 0.079) (Fig. 2b). Those with experience of intravenous drug use were more likely to access HIV testing via the telemedicine than the face-to-face service (3.5% versus 0.4%; *P* = 0.026) (Fig. 2c). During the first and second waves of the COVID-19 pandemic in Thailand, more TGW individuals accessed HIV testing via the face-to-face service than the telemedicine service (63.9% versus 28.1%), which was reversed in the third and fourth waves (36.1% versus 71.9%; *P* < 0.001) (Fig. 2d). In addition, TGW individuals who had never undergone HIV testing accessed HIV testing via the face-to-face service more often than the telemedicine service (27.3% versus 16.4%; *P* = 0.007) (Fig. 2e).

In terms of the reasons for accessing the services at MPlus, TGW individuals who wanted to know their HIV status or had a high risk of HIV infection were more likely to use the face-to-face service than the telemedicine service (50.0% versus 42.3% and 12.0% versus 10.1%, respectively; *P* = 0.044) (Fig. 2f).

### The risk of being HIV-positive during the COVID-19 pandemic adjusted for the type of HIV testing service

According to the univariable logistic regression analysis results in Table 2, the risk of being HIV positive when accessing the face-to-face or telemedicine service was the same (odds ratio [OR] = 1.11; 95% CI = 0.49–2.48; *P* = 0.803). Meanwhile, reactive STIs (OR = 6.29; 95% CI = 2.55–15.50; *P* < 0.001), having a history of drug abuse (OR = 4.88; 95% CI: 1.42–16.74; *P* = 0.012), having sex work experience (OR = 4.80; 95% CI = 1.55–14.82; *P* = 0.006), and never having had an HIV test or having one more than a year ago (OR = 3.99; 95% CI = 1.20–13.22; *P* = 0.024) were all associated with a higher risk of being HIV-positive. Although the risk of being HIV-positive was not significantly different between the types of service, the trend of accessing HIV testing via the telemedicine service noticeably changed between the early and late periods of the COVID-19 pandemic (Fig. 2f). Therefore, to control its influence, the type of service was retained as an adjustment variable in the multivariable model.

**Table 2:** Factors associated with transgender women being HIV positive

Variable	HIV Positive		Univariable Analysis			Multivariable Analysis					
						Full Model^a^			Final Model^b^		
	n/N	(%)	OR	(95% CI)	*P*	aOR	(95% CI)	*P*	aOR	(95% CI)	*P*
Type of service					0.803			0.209			0.329
Face-to-face (ref.)	10/260	3.9	1.00	–		1.00	–		1.00	–	
Telemedicine	16/377	4.2	1.11	(0.49–2.48)		2.36	(0.62–8.99)		1.86	(0.54–6.46)	
Sex worker experience					0.006*			0.106			0.009*
No (ref.)	7/326	2.2	1.00	–		1.00	–		1.00	–	
Yes	6/63	9.5	4.80	(1.55–14.82)		3.22	(0.78–13.31)		5.92	(1.57–22.30)	
Experience with HIV testing					0.024*			0.032*			0.034*
Within the previous year (ref.)	4/247	1.6	1.00	–		1.00	–		1.00	–	
Never or more than 1 year ago	9/146	6.2	3.99	(1.20–13.22)		5.37	(1.15–24.99)		4.05	(1.11–14.77)	
STI result					< 0.001*			0.327			
Non-reactive syphilis (ref.)	16/582	2.8	1.00	–		1.00	–				
Reactive syphilis	8/53	15.1	6.29	(2.55–15.50)		2.98	(0.34–26.55)				
History of drug abuse					0.012*			0.421			
No (ref.)	9/360	2.5	1.00	–		1.00	–				
Yes	4/36	11.1	4.88	(1.42–16.74)		2.09	(0.35–12.56)				
Age (years old)					0.888						
< 25 (ref.)	15/376	4.0	1.00	–							
> 25	11/261	4.2	1.06	(0.48–2.35)							
Relationship status					0.959						
Single (ref.)	11/303	3.6	1.00	–							
With a partner	4/107	3.7	1.03	(0.32–3.31)							
HIV-tested during the window periods					0.269						
No (ref.)	24/614	3.9	1.00	–							
Yes	2/23	8.7	2.34	(0.52–10.58)							
A history of taking PrEP before HIV testing					0.504						
Yes (ref.)	3/118	2.5	1.00	–							
No	11/282	3.9	1.56	(0.43–5.69)							
A history of taking PEP before HIV testing					0.897						
Yes (ref.)	2/66	3.0	1.00	–							
No	11/329	3.3	1.11	(0.24–5.12)							
The reason for HIV testing					0.839						
Others (ref.)	21/554	3.8	1.00	–							
A high risk of HIV exposure	3/70	4.3	1.14	(0.33–3.92)							
Tested during the COVID-19 pandemic time periods					0.263						
First and second waves (ref.)	10/314	3.2	1.00	–							
Third and fourth waves	16/323	5.0	1.58	(0.71–3.55)							

Abbreviations: ref., reference group; OR, odds ratio; aOR, adjusted odds ratio; 95% CI, 95% confidence interval; n, number of HIV positive; N, number of participants; PrEP, pre-exposure prophylaxis; PEP, post-exposure prophylaxis

^*^P-value < 0.05

^a^ All potentially associated factors with *P*-values less than 0.25 in the univariable analyses (i.e., sex worker experience, experience with prior HIV testing, the STI result, and a history of drug abuse), and the type of HIV testing service (face-to-face or telemedicine) were included in a multivariable analysis

^b^ The final multivariable logistic regression model with a backward elimination process

According to the final multivariable model with backward elimination adjusted for the type of service, having sex work experience (adjusted OR [aOR] = 5.92; 95% CI = 1.57–22.30; *P* = 0.009), and never having had an HIV test or being tested more than a year ago (aOR = 4.05; 95% CI = 1.11–14.77; *P* = 0.034) were independently significantly associated with a higher risk of being HIV-positive (Table 2). The final model was selected based on the results of the following best-fit indices: Pearson’s Chi-square goodness-of-fit test (statistical value = 2.88; *P* = 0.579), the Hosmer-Lemeshow goodness-of-fit test (statistical value = 1.90; *P* = 0.754), and the lowest BIC value (dropped by 113.57 to 110.98 compared to the prior model).

## Discussion

Although the face-to-face service is an effective approach for providing information, care, and treatment [19, 20], there were several issues: traveling to the healthcare facility, the staff there being overloaded with work due to insufficient manpower, the worry of HIV status disclosure, and the spread of COVID-19. For comparison, there was a significant decline in the use of public healthcare centers for HIV testing in South Korea from 2019 to 2020 due to the conversion of these centers for COVID-19 testing purposes. This scenario could have amplified concerns among individuals at risk of HIV, causing them to be more hesitant about seeking HIV testing due to potential COVID-19 exposure. Noteworthily, both PLHIV and those at risk of contracting HIV had limited access to telehealth services in South Korea [9]. In this study, we found that during the COVID-19 pandemic, participants were more likely to access HIV testing via the telemedicine service than the face-to-face one. The number of TGW individuals who accessed HIV testing via the telemedicine service markedly increased from 28.1% in the first and second waves of COVID-19 to 71.9% in the third and fourth waves (*P* < 0.001) whereas the opposite was true for the face-to-face service. The reason for this trend could have resulted from the advantages of the telemedicine service (offering an appointment for HIV testing online and performing the test at an affiliated hospital nearby) making it more convenient and safer than the face-to-face service during the pandemic. We also found that the telemedicine service was not associated with an increased risk of being HIV-positive among the TGW participants. Thus, it is an excellent means of reaching out to TGW and other key populations who are unaware of their HIV status, especially those worried about confidentiality during a face-to-face appointment.

Although the numbers of TGW individuals with sex work experience accessing the telemedicine and face-to-face services were similar, their risk of being HIV-positive was six-fold higher than those who had never been sex workers. Despite the HIV testing coverage of sex workers in Thailand being over 60% [21], they comprise a key population with a high risk of HIV infection, and especially, TGW sex workers have a higher risk of contracting HIV than cisgender sex workers [22]. Thus, offering a more convenient channel for HIV testing could play an important role in regular testing for HIV infection.

Our results reveal that the TGW individuals who had not previously undergone HIV testing were more likely to do so via the face-to-face service at the stand-alone center or MVCT than the telemedicine service (27.3% versus 16.4%). On the contrary, Phanuphak et al. (2018), who focused on TGW residing in Bangkok and Pattaya, revealed that individuals who underwent HIV testing for the first time displayed a preference for the online option (which included receiving counseling and supervised HIV self-testing) over the alternatives such as mixed online pre-test counseling with onsite HIV testing or offline HIV counseling and testing [23]. This difference could be related to trust issues about the process and the reliability of being tested. We found that TGW individuals who had not previously undergone HIV testing or who had been tested longer than a year ago had a 4-fold higher risk of being HIV-positive than those who had undergone testing within the previous year. The Centers for Disease Control and Prevention recommends that individuals between the ages of 13 and 64 years old should undergo HIV testing at least once as part of routine healthcare, while those at high risk should be tested more frequently (at least annually) [24, 25]. Therefore, promoting the telemedicine HIV service could help to reach more high-risk individuals currently unaware of their HIV status and persuade them to undergo routine testing. This could reduce the number of new HIV infections and increase the number of PLHIVs on the ART program, thereby helping to achieve the 95-95-95 global target toward ending AIDS.

We also found that the TGW individuals who used drugs intravenously accessed HIV testing more often via the telemedicine service than the face-to-face service. HIV testing coverage among PWIDs in Thailand from 2017 to 2020 was only 38% [21], which could be due to concerns about confidentiality when accessing the service. Thus, implementing an outreach program and promoting the telemedicine service for HIV testing could reach more of the PWID population and persuade them to be tested and enroll in HIV prevention and treatment programs.

There are some limitations to this study. First, there were some missing data values due to the participants not responding to some questions related to the risk of exposure to HIV and information about the service. Second, we did not collect data about the barriers to accessing HIV testing, quality of service, and client satisfaction with the services. Asking for this information could have unveiled more details about the comparative advantages and disadvantages of the two services. Third, the comparison of accessing HIV testing via both services was conducted on data accumulated during the COVID-19 pandemic. A comparative study before and after the pandemic would certainly highlight the impact of COVID-19 on accessing HIV testing as well as other medical services. Finally, the focus of this study was only on TGW living in Chiang Mai and nearby areas during the COVID-19 pandemic, and thus the results cannot be generalized for the whole country.

## Conclusions

The telemedicine service was more popular than the face-to-face service among TGW in Chiang Mai, Thailand during the COVID-19 pandemic. The telemedicine service, which is an alternative channel to access HIV testing, was not associated with a higher risk of being HIV-positive. Promoting the telemedicine service could help more high-risk individuals who are concerned about confidentiality (especially intravenous drug users) to become aware of their HIV status. Sex work experience and irregular of HIV testing are key risk factors of HIV infection for both telemedicine and face-to-face services and should be monitored.

## Data Availability

Data are however available from the authors upon reasonable request and with permission of Mplus Foundation.

## Abbreviations

aOR: Adjusted odds ratio
ART: Antiretroviral therapy
BIC: Bayesian Information Criterion
CI: Confidence interval
face-to-face: Both stand-alone center and MVCT service
IQR: Interquartile range
MSM: Men who have sex with men
MVCT: Mobile voluntary counseling and testing
PEP: Post-exposure prophylaxis
PLHIV: People living with HIV
PrEP: Pre-exposure prophylaxis
OR: Odds ratio
STI: Sexually transmitted infection
TG: Transgender
TGW: Transgender women
UNAIDS: Joint united nations programme on HIV and AIDS
VCT: Voluntary counseling and testing
WHO: World health organization
